# Specific Image Characteristics Influence Attitudes about Chimpanzee Conservation and Use as Pets

**DOI:** 10.1371/journal.pone.0022050

**Published:** 2011-07-13

**Authors:** Stephen R. Ross, Vivian M. Vreeman, Elizabeth V. Lonsdorf

**Affiliations:** 1 Lester E. Fisher Center for the Study and Conservation of Apes, Lincoln Park Zoo, Chicago, Illinois, United States of America; 2 Committee on Evolutionary Biology, University of Chicago, Chicago, Illinois, United States of America; Texas A&M University, United States of America

## Abstract

Chimpanzees are endangered in their native Africa but in the United States, they are housed not only in zoos and research centers but owned privately as pets and performers. In 2008, survey data revealed that the public is less likely to think that chimpanzees are endangered compared to other great apes, and that this is likely the result of media misportrayals in movies, television and advertisements. Here, we use an experimental survey paradigm with composite images of chimpanzees to determine the effects of specific image characteristics. We found that those viewing a photograph of a chimpanzee with a human standing nearby were 35.5% more likely to consider wild populations to be stable/healthy compared to those seeing the exact same picture without a human. Likewise, the presence of a human in the photograph increases the likelihood that they consider chimpanzees as appealing as a pet. We also found that respondents seeing images in which chimpanzees are shown in typically human settings (such as an office space) were more likely to perceive wild populations as being stable and healthy compared to those seeing chimpanzees in other contexts. These findings shed light on the way that media portrayals of chimpanzees influence public attitudes about this important and endangered species.

## Introduction

Chimpanzees are an endangered species across their native range in equatorial Africa [1] but their domestic use in countries like the United States often does not reflect this important conservation designation. While the trade and commerce of endangered species is usually carefully regulated, chimpanzees are bought and sold on the open pet market without any significant federal legislation to regulate such trade. As such, chimpanzees are found not only in accredited zoos, sanctuaries and research facilities but there are also over one hundred chimpanzees kept as personal pets and as performers for the entertainment and advertising industry where they are often dressed in clothing and trained to perform unnatural acts for the amusement of human consumers.

In 2008, Ross et al. [2] presented survey data that illustrated how the public is less likely to consider chimpanzees as endangered compared to other great ape species. This phenomenon was linked to the prevalent use of chimpanzees in movies, television shows and advertisements, where chimpanzees are often inaccurately displayed. These results were the first to link the manner in which chimpanzees are portrayed in popular media to public attitudes that may influence support for critical in-situ conservation efforts. However, the specific means by which these images affected how people characterize chimpanzees was largely unknown. Furthermore, the degree to which these media portrayals affected public attitudes on domestic use of chimpanzees (i.e. the pet trade) remains unexplored in any empirical way. Here we describe an experimental survey study that aimed to identify the specific characteristics of media representations of chimpanzees that may influence the public's perception of this species.

## Materials and Methods

**Figure 1:**
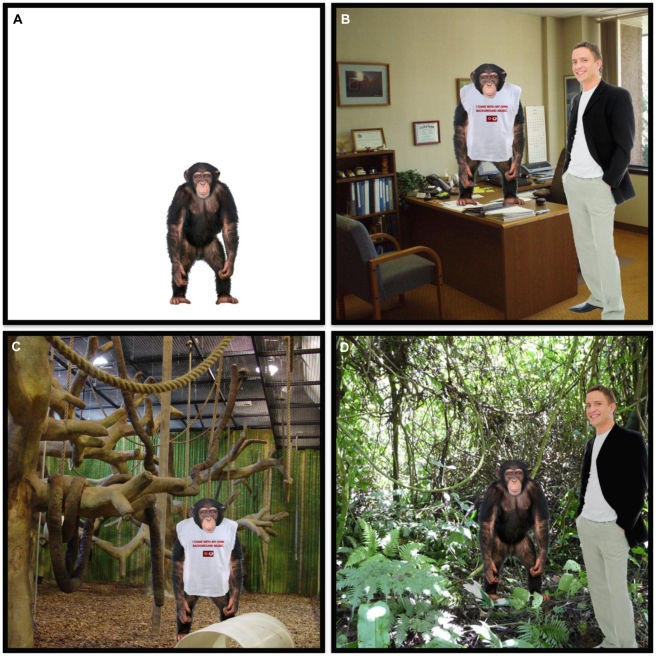
Sample images provided to survey respondents. Detailed legend: Each image was characterized by four variables: clothing (two levels: clothed and unclothed), human presence (two levels: human present, no human present), setting (four levels: a blank, neutral background, an anthropomorphic setting which depicted a typical office setting, a modern zoo setting in which artificial exhibit elements and wire mesh caging were visible, and a naturalistic, jungle bgackground), and media (three levels: photograph, cartoon, pencil line drawing). The images above are a sample of the 48 unique images used. (A) chimpanzee unclothed with no human with a neutral background. (B) chimpanzee clothed with a human in an anthropomorphic setting (typical office space). (C) chimpanzee clothed without a human present in a captive setting. (D) chimpanzee unclothed with a human present in a species-typical (jungle) setting.

In preparation for the survey, a series of composite images were created using Adobe Photoshop version 5.0 (Adobe Systems, San Jose, California, United States of America) by inserting a stock photograph of an adolescent chimpanzee (Figure 1A) onto a background image. Each image was characterized by four variables: clothing, human presence, setting, and media. There were two levels of clothing: images in which chimpanzees were unclothed and those in which the chimpanzee appeared to be wearing a shirt (Figure 1B, 1C). There were two levels of human presence: images in which no human was present and those in which a stock photograph of an adult human male (Figure 1B, 1D) was inserted and appeared to be standing adjacent to, but not interacting with, the chimpanzee. There were four levels of setting: a blank, neutral background (Figure 1A); an anthropomorphic setting which depicted a typical office setting (Figure 1B); a modern zoo setting in which artificial exhibit elements and wire mesh caging were visible (Figure 1C); and a naturalistic, jungle background (Figure 1D). Finally, there were three levels of media type: those in which the image was presented as a photograph, those presented as a cartoon, and those in which the image was presented as a pencil line drawing. In total, there were 48 possible images using all combinations of these four variables. Figure 1(A-D) shows four sample images which illustrate key combinations of these variables.

One of the 48 possible composite images was included as part of each packet of printed materials distributed to study participants by the survey administrator, Responsive Management. Survey respondents (n = 1203) were selected using a random digit dialing procedure to ensure that all sub-samples were representative of the age and gender distributions for the United States. Subjects were blind to the specific topic of study, only knowing that it was a wildlife questionnaire from Lincoln Park Zoo and were contacted via telephone during weekdays between 9:00am and 9:00pm local time. Respondents were asked to view their assigned photograph and subsequently answer a series of questions about chimpanzees. Responses were matched to the characteristics of their assigned photograph to determine potential effects of each variable on public attitudes on this species. Though the survey was much longer, we focus here on responses to two specific questions. Respondents were asked how, after viewing the pictures, they characterized the current conservation status of wild chimpanzee populations. We lumped responses into two categories: those that described the status as declining or endangered and those that described it as healthy or stable. The second question in this analysis asked respondents if they felt they found chimpanzees appealing as pets (agree or disagree). We used a logistic regression model with forward selection to determine which of the four variables (clothing, human presence, setting and media) best predicted responses to these questions.

The survey research protocol was approved by the Lincoln Park Zoo research advisory committee.

## Results

**Figure 2:**
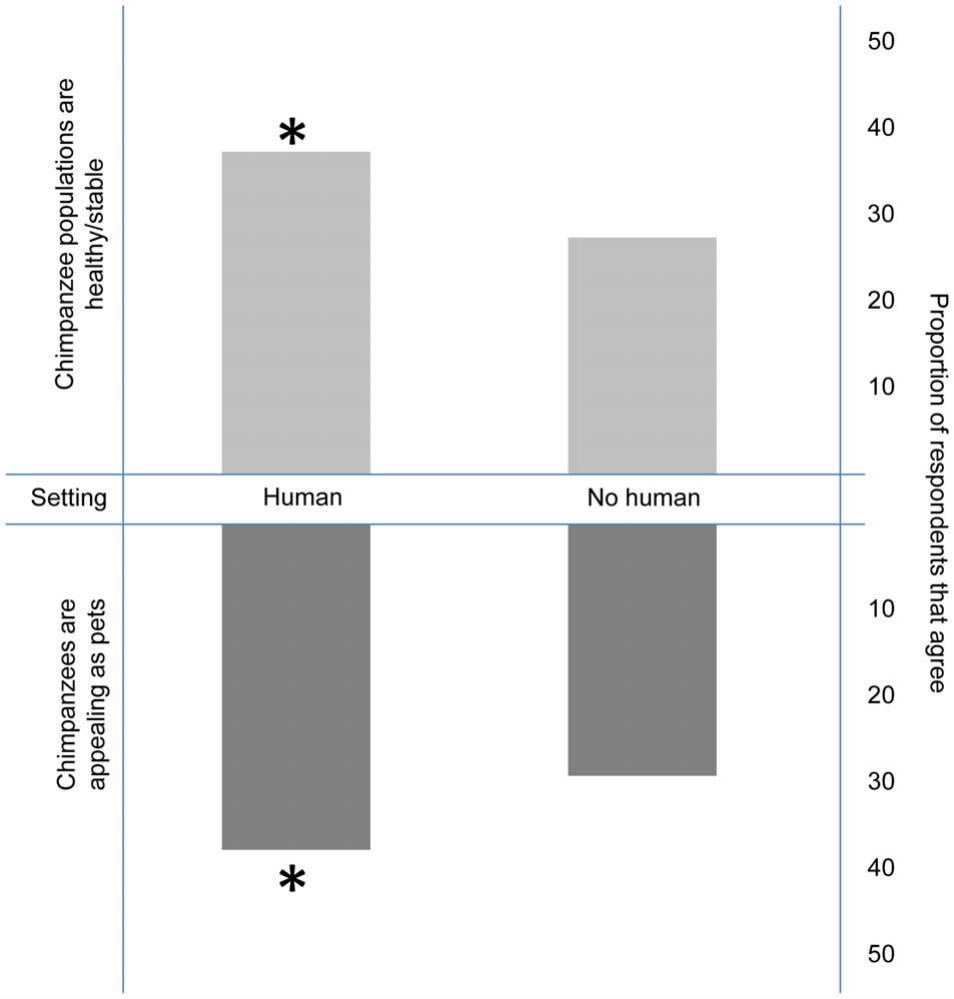
Effect of human presence on perception of chimpanzee population status and appeal as pets. Detailed legend: The top bars display the proportion of survey respondents that agreed that wild chimpanzee populations were healthy/stable when viewing one of two versions of a composite image. In one version of the image, an adult human male was shown standing adjacent to the chimpanzee. In the second version, the chimpanzee was shown without any human present. The bottom bars display the proportion of survey respondents that agreed that chimpanzees were appealing as pets after viewing one of the same images with humans present or not present.

**Figure 3:**
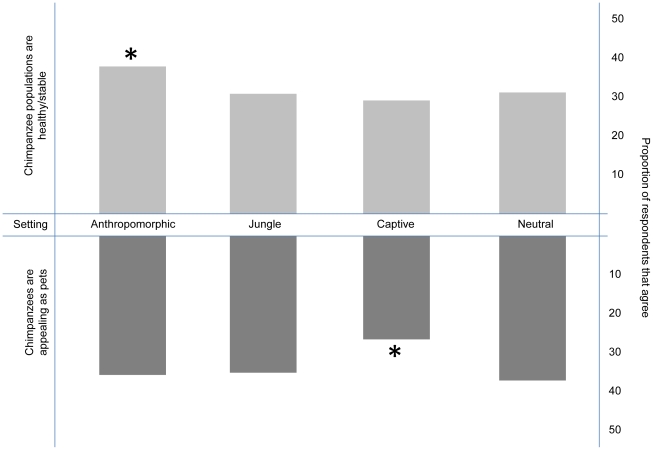
Effect of human presence on perception of chimpanzees as appealing pets. Detailed legend: The top bars display the proportion of survey respondents that agreed that wild chimpanzee populations were healthy/stable when viewing one of four versions of a composite image: those with an anthropomorphic (office setting) background, a jungle background, a captive (zoo setting) background, or a neutral (blank white) background. The bottom bars display the proportion of survey respondents that agreed that chimpanzees were appealing as pets after viewing one of the same images with one of the four above-listed backgrounds.

When respondents were asked to describe the current population status of chimpanzees we found that neither clothing nor medium significantly influenced the distribution of responses, so these factors were excluded from the final regression model. There was a significant main effect of human presence such that those viewing photographs of a chimpanzee standing next to a human, were 35.5% less likely to categorize chimpanzee populations as endangered/declining compared to those viewing photographs with the chimpanzee standing alone (Wald Chi-square = 11.925, p = 0.0006)(Figure 2). The effect of background setting was substantial but not statistically significant (Wald Chi-square = 6.88. P = 0.0757). When we examined independent pairwise contrasts for this variable we found that those viewing images with chimpanzees in an anthropomorphic background (a typical office space) were significantly less likely to categorize chimpanzees as endangered/declining compared to those viewing chimpanzees in neutral, jungle or captive/zoo contexts (Wald Chi-square  = 6.57, p = 0.0104)(Figure 3).

We next investigated how these images could affect public perceptions of chimpanzees as potential pets and analyzed responses of people when asked if having a pet chimpanzee seems appealing. Again, neither clothing nor medium were found to reach criterion for inclusion in the model, but human presence and setting did significantly influence the responses. Those viewing an image of a chimpanzee standing next to a human were 30.3% more likely to agree that a chimpanzee was appealing as a pet than those viewing an image of a chimpanzee standing alone (Wald Chi-square = 9.9886, p = 0.0016)(Figure 2). Likewise, responses from those viewing images of chimpanzees in zoo-like captive settings differed from those viewing chimpanzees in other settings, as they were the least likely to categorize chimpanzees as potentially appealing as pets (Wald Chi-square = 7.92, p = 0.0049) (Figure 3).

## Discussion

The use of chimpanzees in popular media is both prevalent and long-standing in the United States and internationally. Though it is difficult to precisely quantify the prevalence of this practice, we know that chimpanzees have been used as actors and photographic props for at least 90 years in the United States. This use is more than simply a historical practice as at least 59 television advertisements featuring chimpanzee actors have aired since 1986 [3]; most portraying chimpanzees as anthropomorphic caricatures of humans. While there is concern about the animal welfare outcomes for individual chimpanzees involved in the entertainment industry there has been much less focus on how these inaccurate portrayals affect public perception of this species.

The survey data presented here is the first empirical investigation of how inaccurate media representations of endangered animals may affect public perceptions of the species. By using composite images, we were able to maintain consistency between the stimuli and limit potentially confounding variables that may have hindered investigations using authentic media portrayals of chimpanzees. Nonetheless, we recognize the inherent complexity in interpreting these survey results.

Many media portrayals of chimpanzees involve chimpanzees interacting directly with humans. In the composite images, we examined a seemingly more passive relationship such that the chimpanzee was displayed simply standing next to a human. Nonetheless, those viewing such photos were 35.5% more likely to consider wild populations to be stable/healthy compared to those seeing the exact same picture without a human present and 30.3% more likely to find chimpanzees appealing as pets. One explanation for this effect is that viewers are led to believe that such direct associations between humans and chimpanzees are both common and safe. This reasoning is supported by the fact that chimpanzees displayed in zoo settings, where they are assumedly kept safely away from humans, resulted in a significantly lower proportion of respondents who found them appealing as pets. Additionally, images of chimpanzees in close proximity to humans may convey the inaccurate perception that these animals are easily handleable and manageable in ways similar to traditional domesticated species and thereby promote the perception that chimpanzees may make suitable pets. These effects may serve to counteract the efforts of scientific and conservation organizations that have formed strong policy statements condemning the use of primates as pets, citing risks to public health and safety, concerns about animal welfare, and adverse effects on wild populations [4]. Given the dearth of effective legislation regulating the commercial trade in this species, the potential negative effect of these media representations on the primate pet trade are considerable.

Though we have limited the number of potentially influential characteristics in the experimental stimuli, it is difficult to confidently generalize these findings across all possible uses. The chimpanzee image used in this study shows an adolescent, gender-neutral chimpanzee standing bipedally and our analysis cannot quantitatively predict whether a seated chimpanzee, or an adult chimpanzee would have lessened (or strengthened) the effects we found in this analysis. We encourage further investigation of more detailed effects of media representations. Our results provide important groundwork for such investigations by showing that the manner in which chimpanzees are displayed, both in terms of human presence and environmental context, can result in significant effects on public perceptions of that species.

In view of these potential limitations, there remain several implicit reasons why these media effects are important to recognize. First, the data presented here significantly enhance our understanding of how media representations of chimpanzees can have key implications for the way in which the general public perceives this important and imperiled species. The results are reminiscent of well-established marketing effects in the consumer behavior community. The manner in which objects and products are displayed can have powerful impacts in the ways that they are perceived, and consequently the value of objects can be greatly enhanced or degraded by the way in which it is presented [5]. Here, our closest living relative and an endangered species may be devalued, particularly relating to its conservation status, by the way it is represented in the media. Conservation policy strategies are often predicated by leveraging information about the status of wild populations in such a way as to engender public compassion and urgency [6] and we might expect that those that do not perceive chimpanzees to be endangered may be less likely to support important conservation efforts.

It is also important to note that these misportrayals are not only displayed in the United States where the majority of this media is produced, but are frequently distributed internationally, including range countries where wild populations of chimpanzees are under constant threat. The effects we have measured among the American general public might well be evident in other countries, including those in Africa, further increasing the direct risk to conservation efforts by potentially driving an increased demand for the species as pets or lessening the impetus for their protection as endangered species. Though we lack the direct empirical data to support these hypotheses, there may be reason to be concerned about these potential pressures to a species already under considerable threat in its native range.

Finally, though the most inaccurate representations of chimpanzees are found in advertising and popular media outlets, we assert that professionals in the scientific and conservation communities share a responsibility to understand how their own images of this species may be affecting public attitudes. Authentic images of scientists and field workers working closely with chimpanzees may have unintended outcomes that could serve to undermine the important research and conservation messages that individuals and organizations are hoping to convey.

In summary, these data provide novel insight into the important relationships between media portrayals, consumer psychology, animal welfare and conservation. The results of this study have identified a significant influence of an overlooked aspect of popular culture and suggest that important conservation efforts might be hampered by practices previously thought to be harmless. Specifically, these data provide empirical support for scientific and conservation organizations that have called for the end of chimpanzees in the entertainment industry. More broadly, this study demonstrates the potential for objective evaluation of issues in the interface between science and policy.
